# Increased Frequency of Immune Thrombocytopenic Purpura in Coeliac Disease and Vice Versa: A Prospective Observational Study

**DOI:** 10.1155/2018/4138434

**Published:** 2018-04-23

**Authors:** Stefano Bibbò, Claudio Fozza, Giovanni Mario Pes, Rodrigo Rojas, Roberto Manetti, Maria Pina Dore

**Affiliations:** ^1^Department of Clinical, Surgical and Experimental Sciences, University of Sassari, Sassari, Italy; ^2^Baylor College of Medicine, Houston, TX, USA

## Abstract

**Introduction:**

Coeliac disease (CD) and immune thrombocytopenic purpura (ITP) are immune conditions, often associated with other immune disorders. In recent years, increasing attention has been directed towards the association between ITP and CD.

**Aim:**

To investigate the frequency of ITP in CD patients and vice versa and to assess the risk of their association.

**Patients and Methods:**

This was a prospective observational study. All consecutive patients with CD or ITP attending our department were enrolled between January 2016 and December 2017. All patients with CD were screened for ITP and patients with ITP for CD. Odds ratios (ORs) were calculated based on the prevalence in the general population.

**Results:**

Two hundred sixty-one CD patients (212 female, mean age 47 ± 16.1 years) and 32 ITP patients (17 female, mean age 57.8 ± 17.4 years) were enrolled. In the CD cohort, two patients (2/261; 0.8%) reported a previous diagnosis of ITP, compared to the general population; OR was 15.3 (95% CI, 3.82–61.73; *p* < 0.0001). Similarly, in the ITP cohort, two patients (2/32; 6.3%) had a previous diagnosis of CD (OR: 9.89, 95% CI, 2.27–43.16; *p* = 0.0002).

**Discussion:**

A greater frequency of ITP in coeliac patients and vice versa was observed in our study, suggesting an increased risk for patients of developing both disorders.

## 1. Introduction

Coeliac disease (CD) is an immune disorder affecting the small intestine, triggered by the ingestion of gluten in genetically susceptible individuals [1]. The prevalence of CD worldwide has been estimated between 0.5% and 1% [2, 3], in particular, a recent study based on data from the Italian general population reported a prevalence of 0.7% [4]. Coeliac disease is often associated with other autoimmune disorders, maybe through a shared immune-related pathogenesis [5, 6]. We have recently observed an increased prevalence of chronic autoimmune disorders in coeliac patients and more specifically a trend for polyautoimmunity [7].

In the last years, an increasing attention has been directed towards the association between immune thrombocytopenic purpura (ITP) and CD. ITP is a rare acquired thrombocytopenia caused by autoantibodies against platelet antigens [8]; the estimated prevalence in the general population is 0.005% [9]. Several case reports described the coexistence of CD and ITP [10–19]. The first large study designed to investigate this association confirmed an increased risk to develop ITP in coeliac patients and vice versa [20]. Afterwards, two smaller observational studies showed contradictory results. For example, in a cohort of 21 children with ITP from Switzerland, Rischewski et al. were not able to find any case of CD [21], while in a more recent study, Sarbay and colleagues observed one case of CD in a group of 29 ITP patients [22].

The aim of this study was to evaluate the frequency of ITP in a cohort of coeliac patients and conversely, the occurrence of CD in patients affected by ITP.

## 2. Patients and Methods

### 2.1. Study Design

This was a prospective observational study. Consecutive patients with a diagnosis of CD attending the *Gastrointestinal Service* at the Clinica Medica, and patients followed for ITP attending the *Hematological Service* were enrolled in the study from January 2016 to December 2017. Coeliac patients were accurately investigated for a current or previous diagnosis of ITP [23, 24]. Moreover, patients with ITP were studied for CD according to the current guidelines. Briefly, patients were evaluated for serological markers of CD; and if indicated, they were tested for genetic susceptibility and eventually underwent upper endoscopy [25, 26]. Data including age, sex, and body mass index (BMI) were collected from each study subject. In addition, information regarding previous or concurrent illnesses was retrieved by directly questioning and by screening charts. For patients who underwent multiple visits within the study period, only information from the last visit was included.

### 2.2. Setting

The *Clinica Medica* and the *Hematological Service* (Department of Clinical, Surgical and Experimental Sciences—University of Sassari, Italy) are both teaching hospitals in Northern Sardinia.

### 2.3. Eligibility Criteria

Adult patients with a definite diagnosis of CD or ITP available to give their written informed consent were eligible for the study. The diagnosis of CD was established on the basis of a combination of clinical features, biochemical testing, serology markers, and histopathological alterations of duodenal mucosa according to the current international guidelines [25, 26]. The diagnosis of ITP was established according to the *international consensus report* [23].

### 2.4. Ethical Consideration

An Institutional Review Board approval was obtained from the local “*Comitato Etico dell'Azienda Ospedaliero-Universitaria di Sassari*” (Prot. 1892/13).

### 2.5. Statistical Analysis

Patients included in the study were stratified into two independent cohorts (CD patients and ITP patients). In case of coexisting disease, patients were classified according to the disease that appeared earlier. The frequency of ITP was calculated in the CD cohort; similarly, the frequency of CD was estimated in ITP cohort. Odds ratios (ORs) with their 95% confidence intervals (CIs) were calculated to estimate the strength of associations between CD and ITP; the results were considered significant when *p* values were less than 0.05. ORs were calculated based on the prevalence in general population [4, 9]. All the statistical analyses were performed using SPSS statistical package (version 16.0, Chicago, IL).

## 3. Results

A total of 293 patients were enrolled in the study. More specifically, 261 were CD patients (212 female, mean age 47 ± 16.1 years) (Table 1), and 32 were ITP patients (17 female, mean age 57.8 ± 17.4 years). In the CD cohort, two patients were previously diagnosed with ITP (2/261; 0.8%). New cases of ITP were not observed during the study period. Assuming a 0.005% prevalence of ITP in the general population, the calculated OR was 15.3 (95% CI; 3.82–61.73; *p* < 0.0001).

In the group of ITP patients, for two of them (2/32; 6.3%), a diagnosis of CD was made before the study period; the remaining 30 patients were investigated for asymptomatic CD and new cases of CD were not found. The final prevalence of CD in patients with ITP was 6.3%, assuming a 0.7% prevalence of CD in the general population; the OR was 9.89 (95% CI; 2.27–43.16; *p* = 0.0002).

Coexistence of CD and ITP was observed in 4 subjects (3 female and 1 male) (Table 2). Two female developed CD earlier; only one had a history of other autoimmune disorders (rheumatoid arthritis). The last one female was 19 years old, who had a history of chronic ITP in childhood and at the time of an ITP flare, the patient developed CD; furthermore, this patient had concurrent Hashimoto's thyroiditis and a family history of CD and type 1 diabetes. Finally, the only male subject who developed CD had a previous diagnosis of ITP and type 1 diabetes.

## 4. Discussion

In this study, a statistically significant association between CD and ITP was observed. In order to calculate the risk in our studied cohort, Italian data for CD (prevalence 0.7%) [4] and European data (there are no official data on ITP prevalence in Italy) for ITP (0.005%) [9] were used as reference. The risk to develop both diseases in CD or ITP patients was greater than expected in the general population.

Previous case series by Sarbay et al. [22] reported a lower prevalence (3.4%, 1/29), and Rischewski et al. reported the absence of CD (0/21) among ITP patients [21]. The discrepancy observed between our study and the two case series analyzed by Sarbay et al. and Rischewski et al. may be partially explained by sampling bias given the small size of cohorts and the rarity of the disease.

On the other hand, our findings confirmed previous observations reported by Olen et al. [20] who found a positive association between CD and ITP, without difference considering the time of onset. They reported that patients with ITP had a 2.96 risk to develop CD, and similarly, patients with CD had an increased risk to further develop ITP (HR 1.91) [20].

The trend towards polyautoimmunity is a characteristic feature among case reports; more specifically, an association of Hashimoto's thyroiditis or other autoimmune diseases with CD and ITP was reported [11, 12, 16, 17]. Several autoimmune conditions are frequently associated with CD, including autoimmune thyroiditis and type 1 diabetes [7]. Our study confirmed the increased risk to develop CD in patients with ITP and vice versa.

In conclusion, based on our results, the chance to develop ITP in patients with CD or the other way around should be taken into account, especially if additional autoimmune diseases are present.

These observations suggest the need to perform larger studies, in particular to assess environmental and genetic risk factors that predispose to polyautoimmunity.

## Figures and Tables

**Table 1:** Characteristics of the patients enrolled in the study.

Characteristics	CD patients	ITP patients
Number of patients	261	32
Mean age (years)	47.0 ± 16.1	57.8 ± 17.4
Sex (M/F)	49/212	15/17
BMI^∗^ (kg/m^2^)	22.3 ± 3.9	26.4 ± 3.6
History of coexisting CD and ITP		
Absent	259 (99.2%)	30 (93.7%)
Present	2 (0.8%)	2 (6.3%)

^∗^Body mass index.

**Table 2:** Characteristics of patients with coexisting coeliac disease and immune thrombocytopenic purpura.

Patients	Age	Sex	BMI	Earlier disease	Comorbidities	Familiar history
1	50	F	32	CD	Rheumatoid arthritis	Nothing
2	66	F	25	CD	None	Nothing
3	19	F	19	ITP	Hashimoto's thyroiditis	Type 1 diabetes, coeliac disease
4	50	M	25	ITP	Type 1 diabetes	Nothing
